# *CaDHN4*, a Salt and Cold Stress-Responsive Dehydrin Gene from Pepper Decreases Abscisic Acid Sensitivity in *Arabidopsis*

**DOI:** 10.3390/ijms21010026

**Published:** 2019-12-19

**Authors:** Hua-feng Zhang, Su-ya Liu, Ji-hui Ma, Xin-ke Wang, Saeed ul Haq, Yuan-cheng Meng, Yu-meng Zhang, Ru-gang Chen

**Affiliations:** College of Horticulture, Northwest A&F University, Yangling 712100, China; 18848966687@163.com (H.-f.Z.); YaSuLiu@126.com (S.-y.L.); jihuima@126.com (J.-h.M.); W1942399775@126.com (X.-k.W.); saeed_ulhaq@nwafu.edu.cn (S.u.H.); YuanchengMeng07@126.com (Y.-c.M.); Kexuanzhangyumeng@163.com (Y.-m.Z.)

**Keywords:** pepper, dehydrins, *CaDHN4*, abiotic stresses, abscisic acid (ABA) sensitivity

## Abstract

Dehydrins play an important role in improving plant resistance to abiotic stresses. In this study, we isolated a dehydrin gene from pepper (*Capsicum annuum* L.) leaves, designated as *CaDHN4.* Sub-cellular localization of *CaDHN4* was to be found in the nucleus and membrane. To authenticate the function of *CaDHN4* in cold- and salt-stress responses and abscisic acid (ABA) sensitivity, we reduced the *CaDHN4* expression using virus-induced gene silencing (VIGS), and overexpressed the *CaDHN4* in *Arabidopsis.* We found that silencing of *CaDHN4* reduced the growth of pepper seedlings and *CaDHN4*-silenced plants exhibited more serious wilting, higher electrolyte leakage, and more accumulation of ROS in the leaves compared to pTRV2:00 plants after cold stress, and lower chlorophyll contents and higher electrolyte leakage compared to pTRV2:00 plants under salt stress. However, *CaDHN4*-overexpressing *Arabidopsis* plants had higher seed germination rates and post-germination primary root growth, compared to WT plants under salt stress. In response to cold and salt stresses, the *CaDHN*4-overexpressed *Arabidopsis* exhibited lower MDA content, and lower relative electrolyte leakage compared to the WT plants. Under ABA treatments, the fresh weight and germination rates of transgenic plants were higher than WT plants. The transgenic *Arabidopsis* expressing a *CaDHN4* promoter displayed a more intense GUS staining than the normal growth conditions under treatment with hormones including ABA, methyl jasmonate (MeJA), and salicylic acid (SA). Our results suggest that *CaDHN4* can protect against cold and salt stresses and decrease ABA sensitivity in *Arabidopsis*.

## 1. Introduction

Pepper (*Capsicum annuum* L.) is a popular vegetable that is appreciated by people from all over the world. Due to climate change, pepper plant growth and development are affected by different environmental conditions [1]. Pepper is sensitive to various stresses, such as high and low temperature, drought, salinity, heavy metals, and light, during the reproductive stage. These stresses negatively affect pepper germination, growth, loss of photosynthetic pigment, and also affect the reproductive characteristics by causing male sterility, reduced pollination and fertilization, increased premature flower and fruit drop; severe stress can also cause death in pepper plants. Our previous studies reported that the accumulation of DHNs in pepper play an important role in adaption to environmental stresses [2]. A number of studies have shown that dehydrins (DHNs) can prevent membrane lipid peroxidation in cells under oxidative stress, and DHNs have been widely associated with stress tolerance in plants [3,4,5,6].

Plant DHNs are highly hydrophilic proteins, belong to the second sub-family of the late embryogenesis abundant protein family (LEA), that accumulate during embryogenesis and respond to various stress conditions. Generally, these are hydrophilic proteins and contain three conserved motifs: the K, Y, and S fragments [7,8,9]. According to the conserved domains, DHNs are sub-divided into five categories: YnSKn, Kn, SKn, YnKn, and KnS [10,11,12,13,14].

Recent studies showed that DHNs have a characteristic irregular structure that can effectively resist freezing [15]. At low intracellular water potential, DHNs adsorb water molecules and act as osmotic regulators [16]. DHNs are proteins that are closely related to plant growth and development, besides their role in stress responses and distribution in different plant organs. Recently, many DHNs have been identified in plants, such as *Arabidopsis thaliana* [17], *Citrus unshiu* [18], maize [19], and *Vigna radiata* [20]. In potato, *DHN10* accumulated in stems, tubers, young leaves, and flowers [21], similarly, in tobacco, KnS-DHNs were expressed in pollen [22]. In *A. thaliana*, DHNs *ERD14* and *LTI29* were distributed in root tips [16], while in rubber, *HbDHN1* and *HbDHN2* were expressed in leaves, stem, bark, and flowers [23].

Many studies have shown that abscisic acid (ABA) played an important role in regulating genes involved in the plant growth, development, and adaptation to environmental factors. For example, YnSKn- and YnKn-type DHNs were induced by ABA treatment. In rice, DHNs *RAB21* [24] and *A. thaliana* DHNs *RAB18*, *ERD14*, and *LTI29* were induced by ABA [16].

DHNs respond to different environmental factors [25]; in cucumber, *Y3SK2* DHNs improved resistance to high temperature and cold stresses [26]. Likewise, in citrus, DHN, *CuCOR1*9, when overexpressed in tobacco, reduced lipid peroxidation at low temperatures [27]. Under salt stress, in banana, overexpression of *MusaDHN-1* improved the ability of plants to resist oxidative and salt stresses [28]. In wheat, YnSKn-type DHNs, *TaDHN1*, *TaDHN2*, and *TaDHN3* were up-regulated by treatment with 200 mM NaCl [29], and in wheat, DHN *WCOR410* maintained the stability of plasma membrane structure under drought stress [30], while in tomato, *SbDHN1* improved drought resistance [31].

Thus, we speculate that *CaDHN4* plays a crucial role in pepper defense under different abiotic stress conditions. This study aimed to determine the functional regulation of *CaDHN4* under cold and salt stresses, and sensitivity to ABA. We performed the sub-cellular localization analysis. Furthermore, under cold- and salt-stress conditions, we characterized the *CaDHN4* function through virus-induced gene silencing (VIGS) in pepper, and overexpression in *Arabidopsis.* This study provides valuable information regarding the function of this significant gene family in pepper and other important crops.

## 2. Results

### 2.1. Sub-Cellular Localization of CaDHN4

**Figure 1:**
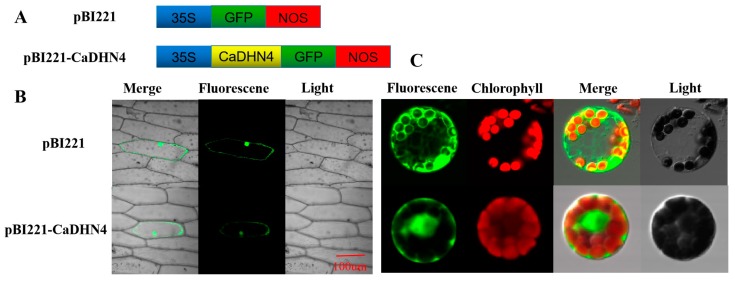
The sub-cellular localization of pepper *CaDHN4*. *(***A**) Schematic diagram of pBI221-*CaDHN4* vector. (**B**) Sub-cellular localization of *CaDHN4* in onion epidermal cells. (**C**) Sub-cellular localization of *CaDHN4* in pepper leaf protoplasts.

The physico-chemical properties of *CaDHN4* genes in pepper are presented in Table S1, with which the localization of CaDHN4 in the nucleus of the cell was predicted. To confirm the sub-cellular localization of *CaDHN4* in pepper, we constructed fusion expression vector of pBI221-*CaDHN4*-GFP through the fusion of the coding-sequence of *CaDHN4* in-frame with GFP (Figure 1A). The fusion constructs pBI221-*CaDHN4* and positive control pBI221 were transiently transformed into pepper leaf protoplasts and onion epidermal cells. By visualizing the GFP signal, we found that *CaDHN4* was expressed in the nucleus and membrane of pepper protoplast cells and onion epidermal cells transfected with the *CaDHN4-*GFP gene fusion construct (Figure 1B,C). This GFP signal indicated that *CaDHN4* is located in the cell nucleus and membrane (Figure 1).

### 2.2. GUS Histological Assay

**Figure 2:**
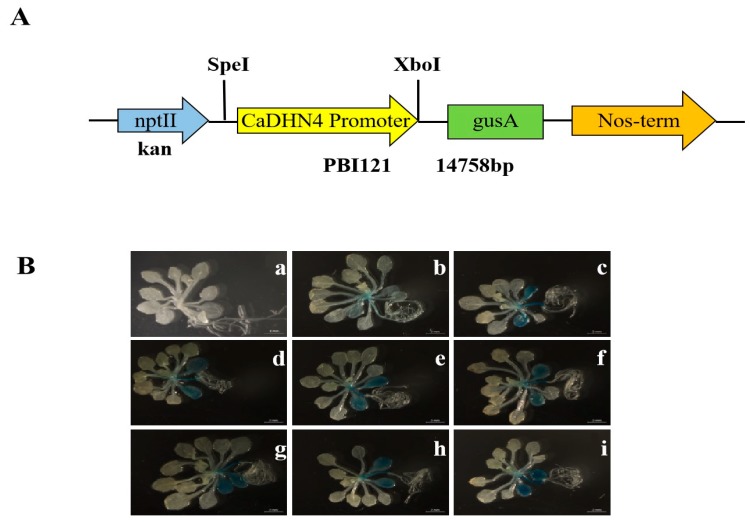
Analysis of *CaDHN4* gene expression patterns. (**A**) Schematic diagram of pBI121::*CaDHN4*-GUS. (**B**) Analysis of GUS activity in transgenic *Arabidopsis* expressing the *CaDHN4* promoter under different stress conditions. (a) Wild type; (b) transgenic *Arabidopsis* under normal temperature; (c) transgenic *Arabidopsis* under low temperature for 4 °C (24 h); (d) 100 mM NaCl (24 h); (e) 250 mM mannitol (24 h); (f) drought treatment (6 h); (g) 100 μM methyl jasmonate (MeJA) (24 h); (h) 100 μM salicylic acid (SA) (24 h); (i) 50 mg/L abscisic acid (ABA) (24 h).

For GUS histological assay, *CaDHN4* promoter region was cloned into the vector pBI121 to construct the plasmid *CaDHN4pro:GU*S (Figure 1 and Figure 2A); the *CaDHN4pro:GUS* gene in transgenic plants were expressed under low temperature, salt, mannitol, drought, methyl-jasmonate (MeJA), salicylic acid (SA), and abscisic acid (ABA) hormone treatments (Figure 2B). The results indicated that *CaDHN4* plays an important role in the regulation of the plant response to diverse environmental stresses.

### 2.3. Virus-Induced Gene Silencing (VIGS) of CaDHN4 Reduces Salt-Stress Tolerance

To further verify the function of *CaDHN4* in response to cold stress, virus-induced gene silencing (VIGS) was performed in pepper cold resistant cultivar “P70.” One month later, we found that the infected pTRV2-phytoene desaturase (PDS) plants were photo-bleached (Figure S1A), which showed that the virus had successfully induced gene silencing in the pepper. The transcript levels were verified by qRT-PCR (Figure S1B). The results showed that the expression of *CaDHN4* was reduced by 90%, implying that *CaDHN4* was successfully silenced through VIGS in pepper.

**Figure 3:**
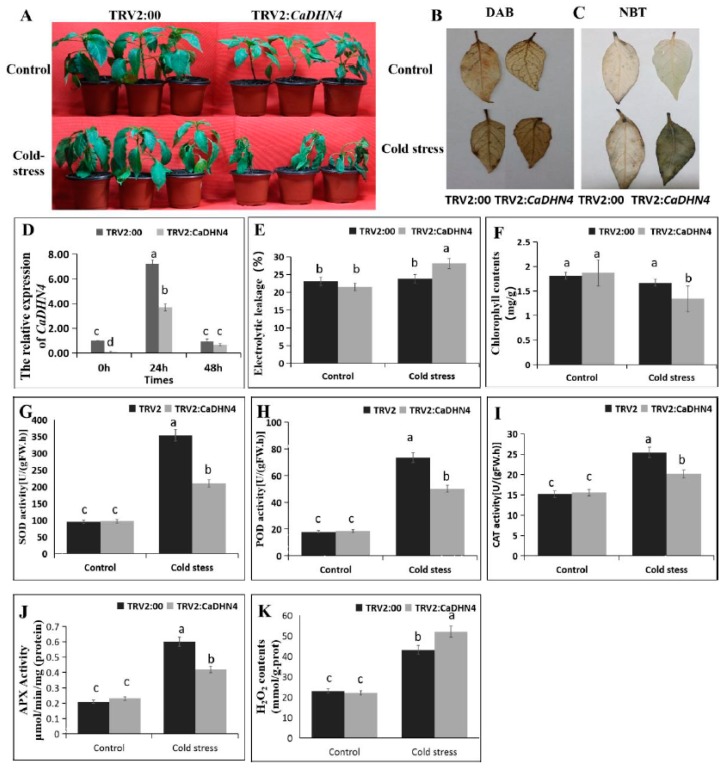
The phenotypes and analysis of *CaDHN4* expression in gene-silenced pepper plants under cold stress. (**A**) The phenotype of silenced pepper plants under cold stress of 3 days. (**B**) 3,3’-Diaminobenzidine (DAB) staining. (**C**) Nitro-blue tetrazolium (NBT) staining. (**D**) The relative expression of *CaDHN4* in silenced pepper plants under cold stress. (**E**) Electrolytic leakage rate. (**F**) Chlorophyll content. (**G**) Superoxide dismutase (SOD) activity. (**H**) Peroxidase (POD) activity. (**I**) Catalase (CAT) activity. (**J**) Ascorbate peroxidase (APX) activity. (**K**) H_2_O_2_ contents. Mean and S.D. values were obtained from three independent experiments, with 3 plants per experiments. The different small letters (a–c) indicate significant differences at *p* ≤ 0.05.

After 3 days’ cold treatment at 4 °C, the pTRV2:*CaDHN4* seedlings showed more aggravated stress symptoms than pTRV2:00 plants (Figure 3A). By analyzing the relative expression of *CaDHN4,* we found that the expression in pTRV2:00 plants increased by almost six-folds, compared to the control plants after 24 h of low temperature treatment. However, the expression of pTRV2:*CaDHN4* plants were only increased by four-fold, where the expression in pTRV2:*CaDHN4* plants were lower than pTRV2:00 plants (Figure 3D). The relative electrolyte leakage and total chlorophyll content of pTRV2:00 and pTRV2:*CaDHN4* plant leaves under cold stress were observed. The relative electrolyte leakage was higher (~15%) in pTRV2:*CaDHN4* plants as compared to pTRV2:00 plants (Figure 3E), and the total chlorophyll content of pTRV2:00 plants was higher (~30%) than that of pTRV2:*CaDHN4* plants (Figure 3F).

To determine the oxidative burst in pTRV2:00 and pTRV2:*CaDHN4* plants, after cold stress, the contents of H_2_O_2_ and (O_2_^−^) were investigated using a histochemical staining method with 3,3’-diaminobenzidine (DAB) and nitro-blue tetrazolium (NBT), respectively. We found an intense staining in the pTRV2:*CaDHN4* leaves as compared to the pTRV2:00 plants (Figure 3B,C), which showed that reactive oxygen species (ROS) production (H_2_O_2_ and O_2_^−^) were higher in pTRV2:*CaDHN4* plants than the pTRV2:00 plants, in response to cold stress.

Next, we measured the activities of superoxide dismutase (SOD), peroxidase (POD), catalase (CAT), and ascorbate peroxidase (APX) in the pTRV2:*CaDHN4* plants and the pTRV2:00 plants under normal or cold stress conditions. In normal condition, the activity of SOD, POD, CAT, and APX were not significantly different in the control and silenced plants. When subjected to cold stress, a remarkable increase in the activities of SOD, POD, CAT, and APX were observed in the pTRV2:*CaDHN4* plants and pTRV2:00 plants. However, these increases of enzymatic activities were higher in pTRV2:00 plants as compared to the pTRV2:*CaDHN4* plants (Figure 3G–J). To know the ROS scavenging mechanism, we tested the accumulation of H_2_O_2_ in the leaves. The results showed that under cold stress, the pTRV2:CaDHN4 plants had higher H_2_O_2_ contents as compared to the pTRV2:00 plants (Figure 3K).

### 2.4. Virus-Induced Gene Silencing (VIGS) of CaDHN4 Reduces Tolerance to Salt Stress

**Figure 4:**
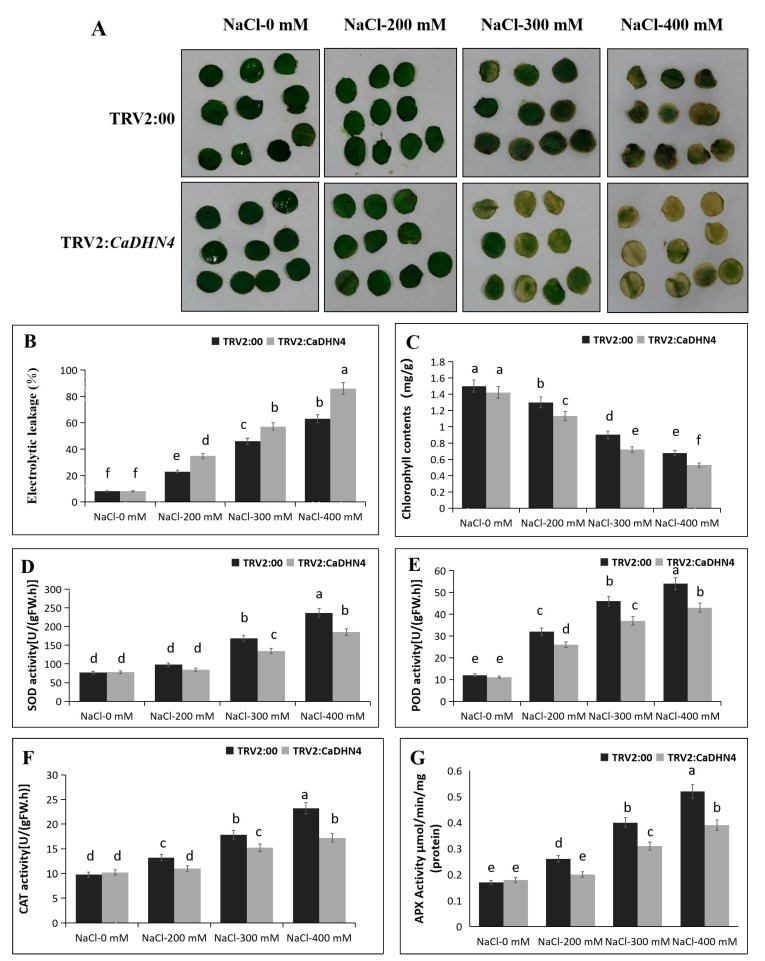
The measurement of physiological indices of *CaDHN4-*gene-silenced pepper plants under salt stress. (**A**) The phenotype of silenced pepper plants under salt stress in detached leaf discs. (**B**) Electrolytic leakage rate. (**C**) Chlorophyll content. (**D**) SOD activity. (**E**) POD activity. (**F**) CAT activity. (**G**) APX activity. Mean and S.D. values were obtained from three independent experiments, with 10 leaves per treatment, respectively. The different small letters (a–f) indicate significant differences at *p* ≤ 0.05.

To investigate the response of *CaDHN4*-silenced plants to salt tress, the pTRV2:00 and pTRV2:*CaDHN4* plants were exposed to 200, 300, and 400 mM NaCl for 3 days. We observed that the color of leaf discs gradually turned yellow with the increase of NaCl concentrations (Figure 4A); the leaf discs from pTRV2:*CaDHN4* plants displayed more necrosis than the pTRV2:00 plants. With the increase of NaCl concentrations, the relative electrolyte leakage was higher in the pTRV2:*CaDHN4* plants as compared to the pTRV2:00 plants (Figure 4B), and the total chlorophyll content of pTRV2:00 plants were higher than the pTRV2:*CaDHN4* plants (Figure 4C).

Then, we measured the activities of SOD, POD, CAT, and APX in pTRV2:*CaDHN4* plants and the pTRV2:00 plants under normal and different NaCl concentrations. In normal condition, the activity of SOD, POD, CAT, and APX were not significantly different between control and silenced plants. While subjected to different NaCl concentrations, a remarkable increase in the activities of SOD, POD, CAT, and APX were observed in pTRV2:*CaDHN4* and pTRV2:00 plants. However, this increase of enzymatic activities was higher in pTRV2:00 plants than the pTRV2:*CaDHN4* plants (Figure 4D–G).

### 2.5. Overexpression of CaDHN4 in Arabidopsis Increases Cold Stress Tolerance

**Figure 5:**
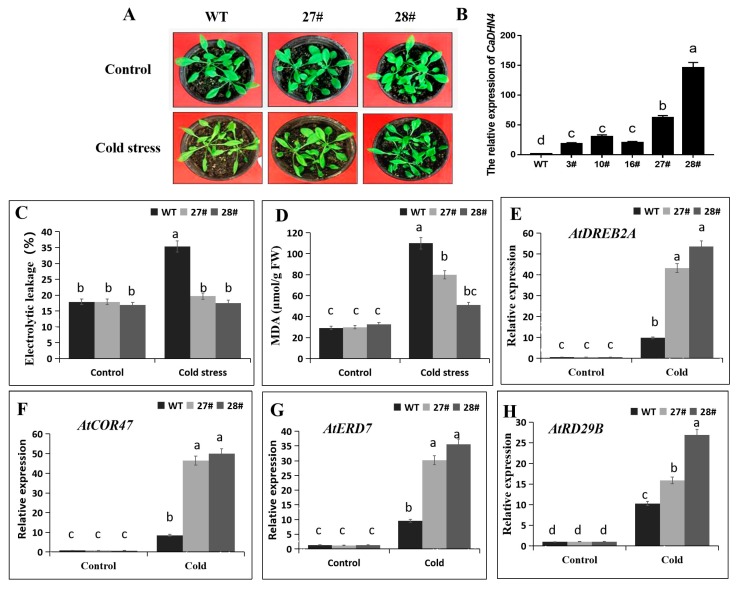
The measurement of physiological indices of *CaDHN4*-overexpressing transgenic and wild-type *Arabidopsis* under cold stress for 3 days. (**A**) Phenotypes of *CaDHN4*-overexpressing transgenic and wild-type *Arabidopsis* under cold stress. (**B**) Expression analysis of *CaDHN4*-overexpressing transgenic *Arabidopsis* plants by qRT-PCR. (**C**) Electrolytic leakage rate. (**D**) Malondialdehyde (MDA) content. (**E**–**H**) qRT–PCR analysis of cold-inducible genes in the CaDHN4 overexpression plants and WT plants in response to cold stress. Mean and S.D. values were obtained from three independent experiments, with 3 plants per treatment. The different small letters (a–d) indicate significant differences at *p* ≤ 0.05.

To explore the response of *CaDHN4*-overexpressing plants to cold stress, we chose five lines of overexpressed transgenic lines of *Arabidopsis*. The relative expression of *CaDHN4* of the lines of (27# and 28#) were significantly higher than three lines (3#, 10# and 16#) (Figure 5B), so the transgenic *Arabidopsis* lines 27# and 28# were selected for the experiments. For cold stress, transgenic *Arabidopsis* and WT plants were exposed to 4 °C for 3 days. The WT plant leaves exhibited a significant higher water loss and morphological changes, whereas, the transgenic plants had green leaves (Figure 5A). To further illustrate the function of *CaDHN4* in regulating tolerance to cold stress, we found that there were no significant difference in the malondialdehyde (MDA) content and relative electrolyte leakage between the WT and transgenic plants under normal growth conditions. However after cold treatment, the WT plants showed an increased level of MDA (3.8 folds) and relative electrolyte leakage (2-folds), as compared to the transgenic plants, where lower levels of MDA (1.6–2.7 folds) and relative electrolyte leakage (1.1 folds) were observed. These data showed that the electrolyte leakage and MDA contents were higher in WT, as compared to the transgenic plants (Figure 5C,D). In addition, to determine whether cold tolerance in *CaDHN4* transgenic plants and interaction of cold-stress-related genes, including *AtCOR47*, *AtDREB2A*, *AtRD29B*, and *AtERD7* assays were performed through qRT-PCR. The expressions of these genes were significantly higher in the *CaDHN4* overexpressed plants than the WT plants (Figure 5E–H). Taken together, these results suggest that the overexpression of *CaDHN4* enhanced cold tolerance in *Arabidopsis*.

### 2.6. Overexpression of CaDHN4 in Arabidopsis Increases Salt Stress Tolerance

**Figure 6:**
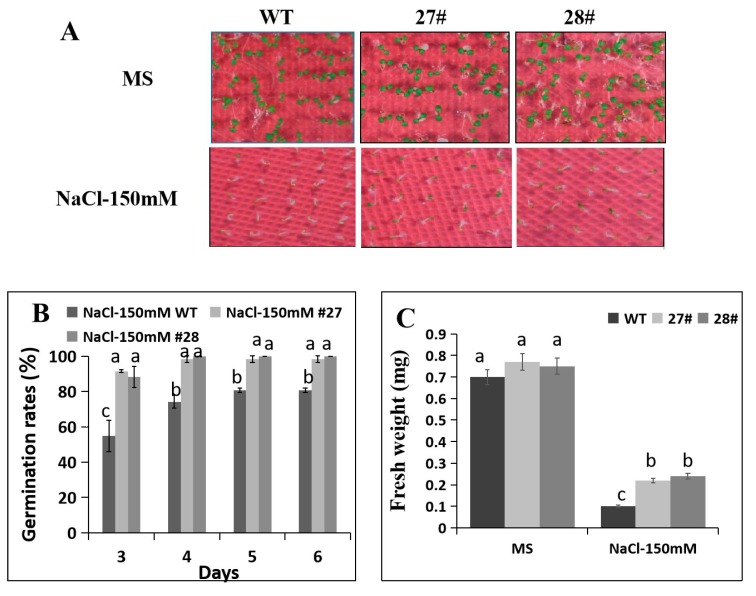
Analysis of salt resistance of *CaDHN4*-overexpressing transgenic and wild type *Arabidopsis*. (**A)** Phenotypes of *CaDHN4*-overexpressing plants under salt stress. (**B**) The seed germination rate of *CaDHN4*-overexpressing transgenic and wild type *Arabidopsis* under 150 mM NaCl treatment. (**C**) The fresh weight of *CaDHN4*-overexpressing transgenic and wild type *Arabidopsis* under 150 mM NaCl treatment. Mean and S.D. values were obtained from three independent experiments, with 30 seeds per treatment. The different small letters (a–c) indicate significant differences at *p* ≤ 0.05.

In order to determine the effect of *CaDHN4* overexpression on salt-stress tolerance, the transgenic and WT *Arabidopsis* plants at different stages of development, namely seed germination, post-germination growth, and mature plants, were exposed to salt stress. We first examined the seed germination rates and post-germination primary root growth of transgenic and WT seeds on 1/2 MS medium or 1/2 MS medium containing 150 mM NaCl. Transgenic plants had the higher seed germination rates than the WT (Figure 6B). To further illustrate the function of *CaDHN4* in regulating tolerance to salt stress, on the third day, we found that the seed germination rates of transgenic plants were higher than WT plants with 150 mM NaCl (Figure 6B). Next, we measured the fresh weight and found that under salt stress, the fresh weight of transgenic plants were higher (~50–52%) than the WT plants with 150 mM NaCl (Figure 6C).

**Figure 7:**
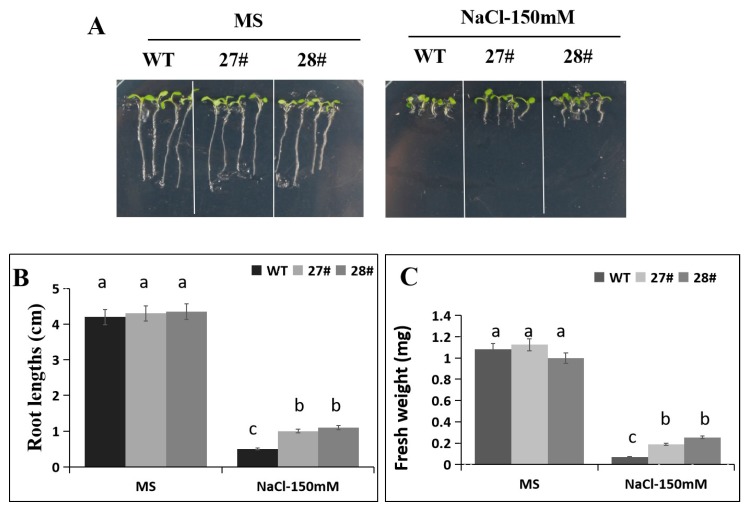
The root lengths of *CaDHN4*-overexpressing transgenic plants and wild type *Arabidopsis* under salt stress. (**A**) The phenotypes of different transgenic lines and WT under control (MS) or NaCl treatment. (**B**) The root lengths of *CaDHN4*-overexpressing transgenic plants and wild type *Arabidopsis* under 150 mM NaCl treatment. (**C**) The fresh weight of *CaDHN4*-overexpressing transgenic plants and wild type *Arabidopsis* under 150 mM NaCl treatment. Mean and S.D. values were obtained from three independent experiments, with 4 plants per treatment. The different small letters (a–c) indicate significant differences at *p* ≤ 0.05.

According to the observations of post-germination primary root growth under salt stress, no significant difference in the root lengths were noticed in the control conditions (0 mM NaCl) between the WT and transgenic plants. However, the root lengths of transgenic plants were higher (~40–60%) than the WT plants with 150 mM NaCl (Figure 7A,B). Next, we also measured the fresh weight and found that under salt stress, the fresh weight of transgenic plants were higher (~50–70%) than the WT plants with 150 mM NaCl (Figure 7C).

**Figure 8:**
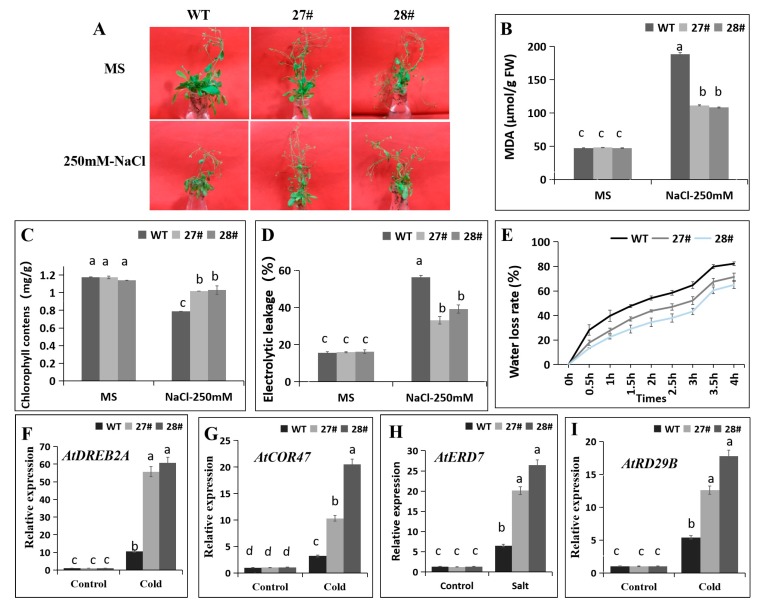
The measurement of physiological indices of *CaDHN4*-overexpressing transgenic plants and wild type *Arabidopsis* under salt stress at mature stage of growth. (**A**) The phenotypes of *CaDHN4*-overexpressing transgenic plants and wild type *Arabidopsis* under salt stress at mature stage. (**B**) MDA content. (**C**) Chlorophyll content. (**D**) Electrolytic leakage. (**E**) Water loss rate.(**F–I)** qRT-PCR analysis of salt-inducible genes in the *CaDHN4* overexpression plants and WT plants in response to high salinity. Mean and S.D. values were obtained from three independent experiments, with 3 plants per treatment. The different small letters (a–d) indicate significant differences at *p* ≤ 0.05.

We subsequently elucidated that overexpression of *CaDHN4* in *Arabidopsis* improved the salt tolerance after 12 h exposure to 250 mM NaCl. We found that after the salt stress, the WT plants exhibited severe wilted leaves phenotypes, while no evident wilting symptoms were observed for the *CaDHN4* overexpressed *Arabidopsis* lines (Figure 8A). Under 250 mM NaCl treatments for 12 h, the transgenic plants showed lower (~40–42%) MDA content and (~25–30%) lower relative electrolyte leakage as compared to WT plants (Figure 8B,D). In contrast, the leaf chlorophyll content of the transgenic plants were higher (~13–14%) than the WT plants (Figure 8C). Through measurement of water loss rate, the transgenic plants showed lower rates of water loss than the WT plants, indicating that *CaDHN4* transgenic plants have an increased water retention capacity (Figure 8E). In addition, qRT-PCR analysis was performed to determine the expression of salt-stress responsive genes, such as *AtCOR47*, *AtDREB2A*, *AtRD29B*, and *AtERD7*. Salt stress induced the expression of these genes in both the WT and *CaDHN4* overexpressed *Arabidopsis* plants as compared to non-treated plants (Figure 8F–I). These marker genes expressed more in the leaves of *CaDHN4* transgenic lines than the WT *Arabidopsis* plants. These data suggest that overexpression of *CaDHN4-*enhanced tolerance to salt stress in *CaDHN4* overexpressed *Arabidopsis* plants.

### 2.7. Overexpression of CaDHN4 in Arabidopsis Decreases ABA Sensitivity

**Figure 9:**
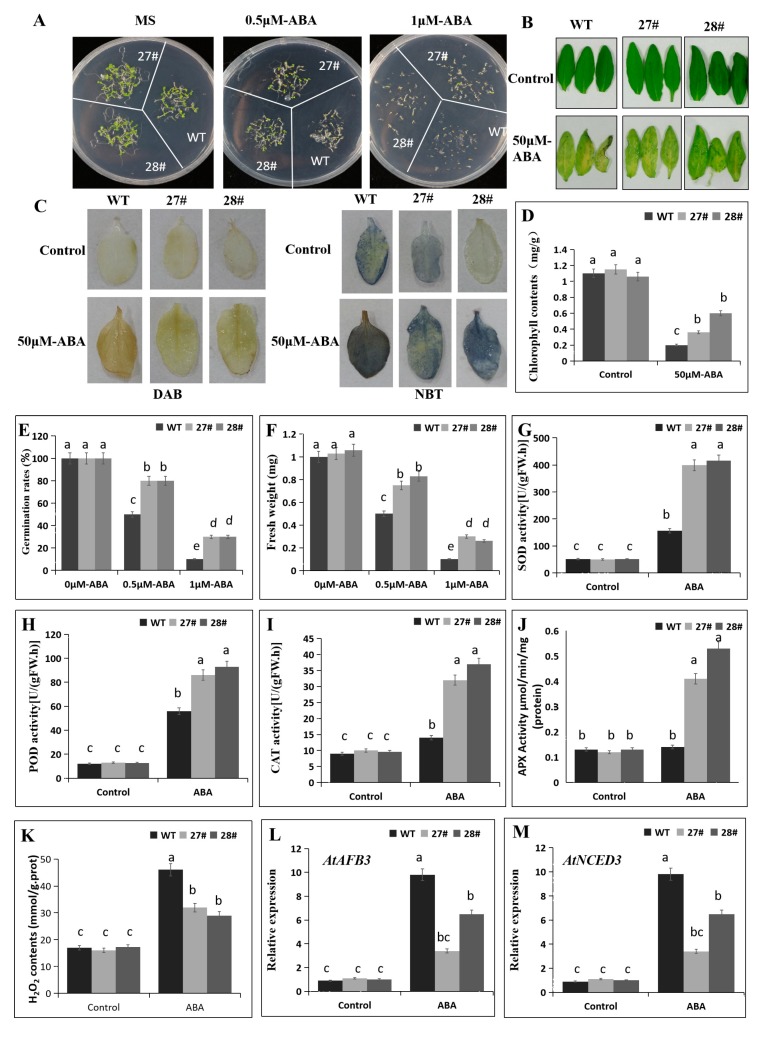
The effect of ABA on seed germination and leaf greening in *CaDHN4*-overexpressing *Arabidopsis* plants. (**A**) The phenotypes of *CaDHN4*-overexpressing plants under ABA treatment. (**B**) Photographs of detached leaves from 4-week-old transgenic *Arabidopsis* and WT plants floated on 1/2 MS medium or 1/2 MS medium containing 50 μM ABA for 3 days. (**C**) DAB and NBT staining. (**D**) Chlorophyll content of *CaDHN4*-overexpressing transgenic and wild type *Arabidopsis* plants under 1/2 MS medium or 1/2 MS medium containing 50 μM ABA. (**E**) The seed germination rate of *CaDHN4*-overexpressing transgenic and wild type *Arabidopsis* plants under different concentrations of ABA. (**F**) The fresh weight of *CaDHN4*-overexpressing transgenic and wild type *Arabidopsis* plants under different concentrations of ABA. (**G**) SOD activity. (**H**) POD activity. (**I**) CAT activity. ( **J**) APX activity. (**K**) H_2_O_2_ contents. (**L**,**M**) qRT-PCR analysis of ABA responsive genes *AtAFB3* and *AtNCED3* in the *CaDHN4* overexpression plants and WT plants after ABA treatment. Mean and S.D. values were obtained from three independent experiments, with 30 seeds and 3 leaves per treatment. The different small letters (a–c) indicate significant differences at *p* ≤ 0.05.

In order to further confirm the function of *CaDHN4*, we overexpressed this gene in *Arabidopsis*. First, we randomly selected five transgenic lines (3#, 10#, 16#, 27#, and 28#) from the T3 generations. Then, we screened these five lines on 0.5 μM ABA, where 27# and 28# performed better than the other lines (Figure S2). In order to determine the role of *CaDHN4* in the ABA response, we initially quantified seed germination rate in response to ABA. The seed germination assay revealed that no significant difference was observed in the seed germination rate between transgenic and WT *Arabidopsis* plants under normal conditions. However, we found that with exposure to 0.5 and 1 μM ABA, the seeds from transgenic plants exhibited much higher germination rates than the WT (Figure 9E). With an increase in ABA concentration, the seed germination rate decreased; the seed germination rates of WT plants were lower than the transgenic plants (Figure 9E). Under 0.5 and 1 µM ABA treatments, the fresh weight of transgenic plants were higher than WT plants (Figure 9F). We further investigated the ABA sensitivity of the *CaDHN4*-overexpressing transgenic lines at the mature stage by floating the *Arabidopsis* leaves on 1/2 MS liquid medium supplemented with 50 μM ABA. As shown in Figure 9B,D, WT plants almost became yellow after 3 days of treatment. The chlorophyll content also indicated that the WT plants lost approximately 90% of the chlorophyll, as compared to the leaves that were not treated with ABA. In contrast, in the transgenic *Arabidopsis* plants’ leaves, yellow areas were lower than those in the WT plants, and lost only 40–60% of the chlorophyll contents, compared to the leaves without ABA treatments.

ABA treatment induced the excessive accumulation of ROS, which led to the oxidative stress. To know whether the ABA sensitivity is correlated with the ROS levels, the accumulation of hydrogen peroxide (H_2_O_2_) and superoxide radical (O^2−^) were measured through DAB and NBT staining, respectively (Figure 9). Under normal growth conditions, there was no significant difference in the ROS accumulation in the overexpressed lines and the WT plants, as shown by the comparable light color of DAB- and NBT-stained leaves. After the exposure to 50μM ABA, we found that the blue or brown colors were more intense in both the leaves of transgenic lines and WT plants, indicating more accumulation of ROS. However, the *CaDHN4-*overexpression lines accumulated less H_2_O_2_ and O^2-^ than the WT plants (Figure 9C).

Next, we measured the activities of three main antioxidant enzymes, i.e., SOD, POD, and CAT in *CaDHN4* overexpressed lines and the WT plants under normal and/or 50 μM ABA conditions (Figure 9G–J). No significant differences were observed for the activities of these three antioxidant enzymes under normal conditions. However, when subjected to 50 μM ABA, a remarkable increase in the activities of SOD, POD, CAT, and APX were observed in both the transgenic *Arabidopsis* lines and WT plants. However, the higher enzymatic activities were more pronounced in the overexpression *Arabidopsis* lines as compared to the WT plants. In addition, we also measured the H_2_O_2_ contents under ABA treatment through NBT and DAB staining. No significant difference in H_2_O_2_ contents under normal conditions were noticed; however, under 50 μM ABA, the H_2_O_2_ contents in overexpressed *Arabidopsis* lines were lowered than the WT plants (Figure 9K).

Then, qRT-PCR analysis was performed to examine how the *CaDHN4* overexpression affects the ABA signaling and biosynthesis. Treatment with 50 μM ABA highly induced the ABA responsive genes such as *AtAFB3* and *AtNCED3* in 3 week-old *CaDHN4* overexpressed *Arabidopsis* and WT plants. The expression levels of these genes were lower in the *CaDHN4* overexpressed *Arabidopsis* than the WT plants (Figure 9L,M). This suggests that the *CaDHN4* gene plays an important role in ABA-induced gene expression.

**Figure 10:**
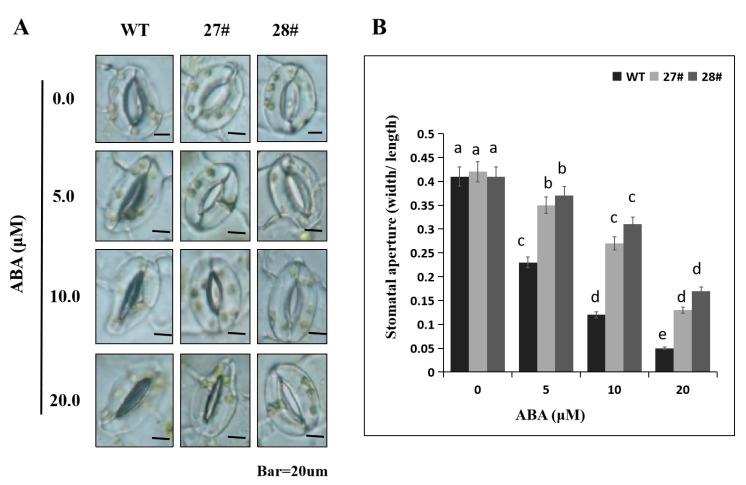
Alterations in stomatal aperture of *CaDHN4* transgenic *Arabidopsis* in response to ABA treatments. (**A**) Stomatal aperture of *CaDHN4* transgenic and WT plants in response to ABA. (**B**) Stomatal aperture values. Mean and SD values were obtained from three independent experiments. The different small letters (a–e) indicate significant differences at *p* ≤ 0.05.

Next, we measured the ABA-induced stomatal conductance. Leaves from 3-week-old *Arabidopsis* plants were incubated in stomatal opening solution and treated with different concentrations (0, 5, 10, and 20 μM) of ABA for 2 h, and then we measured the stomatal aperture. We found that no obvious difference in stomatal aperture between WT and transgenic *Arabidopsis* lines under 0 μM ABA (Figure 10A). However, after treatment with ABA for 2 h, the stomatal apertures of the WT plants were smaller (~30–70%) than the *CaDHN4*-overexpressed transgenic *Arabidopsis* plants (Figure 10B). These results showed that *CaDHN4* decreased sensitivity to ABA.

## 3. Discussion

DHNs have a protective effect on the low temperature and can maintain the stability of cell membranes by preventing the lipid peroxidation and also scavenging of the reactive oxygen species [32]. To understand the regulatory mechanism of *CaDHN4* under different stresses, virus-induced gene silencing (VIGS) of *CaDHN4* was performed in pepper. Under cold stress, there was more serious wilting in the pTRV2:*CaDHN4* silenced pepper plants than the pTRV2:00 pepper plants. Electrolyte leakage and chlorophyll contents in leaves are often used as indicators of plant membrane injury under abiotic stresses [33]. MDA, the product of lipid peroxidation caused by reactive oxygen species (ROS), are used to evaluate ROS-mediated injuries in the plants [34]. The electrolyte leakage and chlorophyll contents also showed that in the pTRV2:*CaDHN4* plants, membrane damage and leaf senescence were more serious under low temperature stress. Thus, MDA content and electrolyte leakage were measured to assess the role of *CaDHN4* in the overexpressed *Arabidopsis* plants, which reduced the membrane injury under cold and salt stresses. The level of electrolyte leakage in the WT were significantly higher than the transgenic *Arabidopsis* plants under cold and salt stresses (Figure 5 and Figure 8), and the MDA content in the WT plants were significantly higher than the transgenic plants under cold and salt stresses. Silencing of *CaDHN4* in pepper, the electrolyte leakage displayed an opposite pattern. These results were consistent with those described by Chen et al. [35].

The DAB and NBT staining results showed more intense staining in the pTRV2:*CaDHN4* pepper plants, as compared to the pTRV2:00 pepper plants. These data indicated that silencing of *CaDHN4* resulted in higher accumulation of hydrogen peroxide and superoxide anions under low temperature stress. To test the regulatory mechanisms of *CaDHN4* and further understand the function of *CaDHN4*, we generated *CaDHN4*-overexpressed *Arabidopsis* plants and determined their responsiveness to cold and salt stresses. Under normal conditions, there were no obvious differences in the plant growth, morphology, and physiological indices between the transgenic *Arabidopsis* and WT plants. After cold stress treatment for 3 days, the transgenic *Arabidopsis* plants grew better than the WT plants. These results indicated that the degree of cell membrane damage and lipid peroxidation of transgenic *Arabidopsis* plants were lower than the WT plants, under low temperature stress. Our results are consistent with the overexpression of maize *ZmDHN2b* in tobacco, which resulted in the higher electrolyte leakage and MDA content in transgenic plants, which were lower than the WT plants under 4 °C treatment [36]. In wheat, overexpression of the acidic dehydrin WCOR410 improved the freezing tolerance in the strawberry leaves [37].

When plants are subjected to abiotic stresses, they accumulate more ROS that may cause oxidative damage to the biomolecules in the plants [38], so the ROS scavenging is closely related to the plants’ tolerance to abiotic stresses. To know the ROS scavenging mechanism, we tested the accumulation of H_2_O_2_. The results showed that under cold stress, the pTRV2:*CaDHN4* pepper plants had higher H_2_O_2_ contents as compared to the pTRV2:00 pepper plants. While, under ABA treatment the overexpressed *Arabidopsis* lines displayed an opposite pattern. In addition, SOD, POD, CAT, and APX as the antioxidant enzymes involved in ROS scavenging, can scavenge H_2_O_2_. We measured SOD, POD, CAT, and APX activities under cold, salt, and ABA treatment. The results showed that under cold stress, the pTRV2:00 pepper plants had higher SOD, POD, CAT, and APX activities than pTRV2:*CaDHN4* silenced pepper plants. In contrast, under ABA treatment, the overexpressed *Arabidopsis* lines had lowered enzymatic activities than the WT plants. These results indicated that silencing of *CaDHN4* reduced the activities of SOD, POD, CAT, and APX activities by inhibiting the expression of antioxidant related genes in vivo, leading to the less accumulation of ROS in plants, resulting in reduced tolerance of plants to cold stress; whereas, the ABA treatment displayed an opposite pattern in *Arabidopsis*.

Under salt stress, the results showed that the germination rate, fresh weight, chlorophyll contents, and root lengths of transgenic plants were higher than the WT plants; whereas, the MDA content and electrolyte leakage of the transgenic plants were lower. These results are in agreement with studies in other plants such as wheat, where overexpression of *TaDHN1* and *TaDHN3* resulted in the better growth and longer roots than the control plants under salt stress [29]. *CaDHN4*-overexpressed *Arabidopsis* plants displayed tolerance and a better growth under cold and salt stresses. These results demonstrated that *CaDHN4* is a positive regulator of cold and salt stresses tolerance in *Arabidopsis*. The mechanisms responsible for improved cold and salt tolerance in *Arabidopsis* might be due to several beneficial changes at the morphological and physiological level. Additionally, overexpression of *CaDHN4* in *Arabidopsis* resulted in higher seed germination rate than WT plants with exogenous ABA treatment. These data suggested that overexpression of *CaDHN4* in *Arabidopsis* decreased the sensitivity to exogenous ABA, which endorses the previous findings of Fujita et al. [39].

Taken together, these data showed that *CaDHN4*-overexpressed *Arabidopsis* plants exhibited a decreased lipid peroxidation and membrane injury under salt- or cold-stress conditions than the WT plants. Plants possess a very efficient enzymatic antioxidant defense system to protect cells from oxidative damage by scavenging ROS, such as SOD, POD, CAT, and APX. According to this study, showed that *CaDHN4* gene maintained the stability of cell membranes, prevented lipid peroxidation and lowered the accumulation of reactive oxygen species, thereby playing an important role in low temperature stress tolerance.

The cis-acting elements in promoters play a crucial role in the regulation of gene transcription [40]. Many DHN genes and their promoters have been identified, and their regulatory pathways have been well discovered [27,41,42]. Analysis of GUS activity of the promoter of *CaDHN4* in transgenic *Arabidopsis* under different stress conditions found that low temperature, NaCl, mannitol, drought, MeJA, SA, and ABA treatments, resulted in intense staining in the transgenic plants, as compared to the control plants. These results are consistent with studies on barley *OsDHN1* gene promoter [43], banana *MusaDHN1* promoter [28], and three wheat DHN promoters (*TaDHN1, TaDHN2,* and *TaDHN3*) [29]. These results showed that *CaDHN4* are involved in pathways related to cold and salt stresses.

Overexpression of *CaDHN4* induced the expression of the other stress-related genes including *AtERD7*, *AtCOR47*, *AtRD29B,* and *AtDREB2A* (Figure 9). These genes are involved in the mediation of osmosis and/or oxidative damage. For example, *AtRD29B* encodes a hydrophilic protein that promotes water retention and maintains membrane integrity [44]. *AtDREB2A* is a member of the AP2/ERF family, and plays an important role in cold stress [45]. Increased expression of these genes (*AtERD7*, *AtCOR47*, *AtRD29B,* and *AtDREB2A*) in *CaDHN4* can help plants to cope with the adverse environmental conditions.

We selected lines 27# and 28# for further investigations based on the screening results on 0.5 μM ABA and expression analysis (Figure S2) of randomly selected 5 transgene linesfrom T3 generations. Our selection of two lines for further studies is consistent with Huang et al. [46], Guan et al. [47], and Shi et al. [48]. ABA act as an important phytohormone in the plant responses to abiotic stresses [49], and several studies have shown that ABA sensitivity correlate with stress tolerance in plants [50,51,52]. Our results demonstrated that overexpression of *CaDHN4* in *Arabidopsis* decreased the ABA sensitivity, perhaps through the ABA-mediated signaling pathway. This was supported by the *CaDHN4* promoter: GUS genes in transgenic plants were expressed under ABA hormone treatments (Figure 2B). *CaDHN4* overexpression also resulted in sensitivity to ABA. The germination rate of *CaDHN4* overexpressing lines was higher than the WT plants after exposure to exogenous ABA (Figure 9). Expression of the ABA-responsive genes *AtNCED3* and *AtAFB3* were significantly decreased in the *CaDHN4*-overexpressed *Arabidopsis* lines as compared to the WT plants (Figure 9). These data suggest that *CaDHN4* might participate in the regulation of the expression of ABA biosynthesis-related genes. The results showed that *CaDHN4* expression negatively regulates ABA signaling by decreasing the ABA sensitivity of plant growth and restraining the induced expression of ABA-responsive genes. Based on the results, we found that although the threshold level of transcripts was different, both independent transgenic lines displayed similarly, such as the phenotypic change, the root length, the germinate rate and so on. The two overexpression lines showed different responses in some of the assays, which may be due to the difference in the relative expressions of the two overexpressed lines. The higher the expression, the stronger was the resistance; on the contrary, the lower the expression, the lower was the resistance. It can be seen in the Figure 5A, Figure 6A, and Figure 8A. The results are consistent with Zhai et al. [53], i.e., the higher expression of OE#4 has stronger drought and salt-stress tolerance in *Arabidopsis* than the lower expression of OE#1, and Cao et al. [23], i.e., the higher expression has stronger abiotic stress responses in *Aarabidopsis*. The level of expression may be relative to the position effect resulting gene silencing [54], DNA methylation [55], and so on.

On the other hand, over expression of *CaDHN4* increased the salt tolerance in overexpression lines. We speculate that after overexpression of *CaDHN4* in *Arabidopsis*, it might have induced the other signaling pathways, as the ABA regulatory mechanism is relatively complex. The enhanced salt tolerance in *CaDHN4*-overexpressing *Arabidopsis* plants could be due to direct/indirect regulation of stress-responsive genes through an ABA-mediated pathway. In summary, *CaDHN4* has a role in the ABA-dependent signaling transduction pathways that induce cold and salt stress tolerance in *Arabidopsis*

Taken together, this study provides substantial evidence that this gene might be involved in cold and salt stress tolerance, and decrease ABA sensitivity in *Arabidopsis*.

## 4. Materials and Methods

### 4.1. Plant Materials and Growth Conditions

The *Arabidopsis* Wild type Col-0 and pepper cold resistant cultivar ‘P70′ provided by College of Horticulture, Northwest A&F University, Yangling, China (34°20’N 108°24’E); were used in this study. Pepper plants were grown at 22 °C with 75% relative humidity and no supplemental light. The transgenic *Arabidopsis* plants were grown at 22 °C with 75% relative humidity under long-day (16 h of light at 125 µmol·m^−2^·s^−1^ and 8 h of dark) conditions.

### 4.2. RNA Isolation and qRT-qPCR Analysis

Total RNA was extracted by using the Tiangen RNA extraction kit (TianGen, Xi’an, China), according to the manufacturer’s protocol, and the synthesis of cDNA and qRT-PCR were executed as described by Chen et al [56]. Relative expression levels were analyzed according to the 2^−ΔΔCT^ method [57]. ACTIN2 was used as an internal control. The primer sequences of qRT-PCR are listed in Table S2.

### 4.3. Isolation and Sequence Analysis of CaDHN4

Full-length of *CaDHN4* (519bp) was amplified from the template cDNA of pepper cultivar P70, and *CaDHN4* (CA02g22060) was obtained by pepper genome database (http://peppergenome.snu.ac.kr/). The forward and reverse primers for full-length of *CaDHN4* cDNA sequences were 5′ATGTCGCACTACGAGAACCA3′ and 5′ CTACTAGTGGTGGCCAGTGCC 3′, respectively. PCR products were cloned into the pMD19-T vector (TaKaRa, Dalian, China) and sequenced (Shanghai GeneCore Biotechnologies Co. Shanghai, China).

### 4.4. Promoter Activity Assay Analysis

The gene-specific primers F1(5′-GACTAGTAGATTTTAATTTGCATGTATGAGC-3′ *SpeI* site underline) and R1(5′-CCGCTCGAGCTTTCTGAACTAAGAACTGACCG-3′ *XhoI* site underline) were used to amplify the *CaDHN4* transcription start site, and then linked with cloning vector pMD-19T (Takara), and after re-sequencing, inserted into vector pBI121, and the recombinant plasmid pBI121-CaDHN4-GUS was obtained. For analyzing the promoter activity of *CaDHN4* under abiotic stresses, 3-week-old plants were selected with 4 °C, 100 mM NaCl, 250 mM mannitol, 100 μM MeJA, 100 μM SA, and 50 mg/L ABA for 24 h for GUS staining. The samples were stained in GUS staining buffer at 37 °C for 24 h and de-colored with 75% alcohol, and then viewed and photographed under a microscope.

### 4.5. Subcellular Localization Assays

The gene-specific primers F2 (5′-GCTCTAGAATGTCGCACTACGAGAACCA-3′ *Xba*I site underline) and R2 (5′-CGGGATCCCTAGTGGTGGCCAGTGCC-3′ *Kpn*I site underline) were used to amplify the *CaDHN4* coding region without stop codon (TGA) and then linked with cloning vector pMD-19T (Takara), and after re-sequencing, inserted into vector pBI221, and the recombinant plasmid pBI221-*CaDHN4*-GFP was obtained. The empty vector pBI221-GFP was used as a control. The confocal laser-scanning microscope was used for GFP fluorescence detection (Nikon, Tokyo, Japan).

### 4.6. Virus-Induced Gene Silencing (VIGS) of CaDHN4

To silence *CaDHN4*, virus-induced gene silencing (VIGS) constructs, pTRV1 and pTRV2 vectors (tobacco rattle virus), were used. They were amplified by the gene-specific primers F3 (5′-GCTCTAGAATTGTGTAGTACGGTCAGTTCTT-3′ *Xba*I site underline) and R3 (5′-CGGGATCCCAGTTGAATGGGCTTGGTC-3′ *BamH*I site underline), then linked with cloning vector pMD-19T (Takara), and after re-sequencing, inserted into vector pTRV2, and the recombinant plasmid pTRV2:*CaDHN4* was obtained. *Agrobacterium tumefaciens* GV3101 containing pTRV1 or pTRV2:*CaNDHN4* were injected into pepper plants and plants were grown under conditions as described by Guo et al [58].

### 4.7. Generation of CaDHN4 Transgenic Arabidopsis Plants

Gene-specific primers F4 (5′-GCTCTAGAATGTCGCACTACGAGAACCA-3′ *Xba*I site underline) and R4 (5′-CGGGATCCCTACTAGTGGTGGCCAGTGCC-3′ *Kpn*I site underline) were designed to amplify the coding region of *CaDHN4*, then linked with cloning vector pMD-19T (Takara), and after re-sequencing, inserted into vector pVBG2307, and the recombinant plasmid pVBG2307-*CaDHN4* was obtained. The pVBG2307-*CaDHN4* was introduced into the *Agrobacterium tumefaciens* strain GV3101 by electroporation. Transgenic *Arabidopsis* plants were obtained using the floral dipping methods. Transgenic plants were screened by 1/2MS medium supplemented with 50 mg/L kanamycin, and the T_3_ generation was harvested for further use.

### 4.8. Salt and Cold Stress Tolerance Assays

For salt treatment, the transgenic *Arabidopsis* plants of seed germination rate and root length, and seeds from T_3_ transgenic *Arabidopsis* plants and WT were surface-sterilized and sown on 1/2 MS medium or 1/2 MS medium containing 150 mM NaCl for 1 week: plants were grown with a 16 h light/8 h dark cycle at 22 °C, 30 seeds from each line; at maturity, the 4-week-old WT and transgenic *Arabidopsis* plants were immersed in 250 mM NaCl for 12 h. For silencing of *CaDHN4* through VIGS, the pepper leaf discs of TRV2:00 and TRV2:CaDHN4 were immersed in different concentrations of NaCl solution (0, 200, 300, and 400 mM) with continuous illumination for 3 days, with each treatment containing 10 pepper seedlings [53]. For cold treatment, the overexpressing *Arabidopsis* plants, WT plants, TRV2:00, and TRV2:CaDHN4 plants were treated at 4 °C for 3 days.

### 4.9. Determination of Chlorophyll, MDA, and Relative Electrolyte Contents and Antioxidant Enzyme Activities

The malondialdehyde (MDA) contents were determined following Dhindsa et al [59]; electrolyte leakage contents were measured as described by Dionisio-Sese and Tobita [60]; chlorophyll contents were measured as described by Arkus et al [61]. The activity of peroxidase (POD), superoxide dismutase (SOD), and catalase (CAT) was detected according to Liang et al [62]; the APX activity was assayed as previously described [63]; the H_2_O_2_ content was determined by the method of titanium oxidation with hydrogen peroxide-titanium complex formation [64].

### 4.10. NBT and DAB Staining

To determine the accumulation of hydrogen peroxide (H_2_O_2_) and superoxide (O^2−^) under cold stress, 3,3’ -diaminobenzidine (DAB) and nitro-blue tetrazolium (NBT) staining were performed [65,66]. Each treatment, leaves were collected and cultivated in DAB (1 mg mL^−1^, pH 3.8) or NBT solution (0.1 mg mL^−1^) overnight at 25 °C. After staining, the chlorophyll was removed by soaking in 75% ethanol and boiled for about 15 min. After sufficient bleaching, the images were captured.

### 4.11. ABA Tolerance Assays

Germination assays were performed on seeds from T_3_ transgenic lines and WT plants. Seeds were surface-sterilized and sown on 1/2 MS medium with or without 0.5μM ABA and 1μM ABA supplementation, and the germination rates of at least 30 seeds from each line were recorded daily. Additionally, the chlorophyll contents of detached leaves were measured. For the ABA sensitivity assay, leaves from 4-week-old soil-grown plants from each line were floated abaxial side up in 1/2 MS liquid medium with or without 50 μM ABA for 3 days at room temperature with a 16 h light/8 h dark cycle followed by chlorophyll content measurement [67].

### 4.12. Measurement of Stomatal Aperture in Response to ABA Treatment

For the ABA-induced stomatal aperture measurement, leaves from 3-week-old T3 transgenic and WT plants were incubated in stomatal opening solution (10 mM KCl, 10 mM MES-Tris, pH 6.15, and 50 μM CaCl_2_). After incubation for 2 h, leaves were treated for 2 h with different concentrations of ABA (0, 5, 10, and 20 μM). Then taking pictures with a light microscope and the stomatal apertures were measured using Image J software. Stomatal aperture values are presented as means from at least 50 stomata.

### 4.13. Statistical Analysis

All data were analyzed by one-way ANOVA (Analysis of variance) using the SPSS (Chicago, IL, USA), and the means were compared using Tukey’s HSD (Honest significant difference, Chicago, IL, USA) multiple range test, taking *p* ≤ 0.05 as a significant difference.

## 5. Conclusions

In this study, we isolated a dehydrin gene from *Capsicum annuum* leaves, designated as *CaDHN4*. To verify the functional role of *CaDHN4* in abiotic stresses tolerance, *CaDHN4* was overexpressed in *Arabidopsis* and silenced in pepper plants through VIGS. Silencing of *CaDHN4* decreased pepper cold and salt tolerance, but *CaDHN4*-overexpressing in *Arabidopsis* enhanced cold-and salt-stress tolerance. Moreover, overexpression of *CaDHN4* in *Arabidopsis* decreased the ABA sensitivity. The level of expression may be relative to the position effect, resulting in gene silencing and DNA methylation. In the future experiments, we will authenticate it. Taken together, the results of our study showed that *CaDHN4* might act as a positive regulator of cold- and salt-stress tolerance. This study will provide further insight for functional analysis of DHN genes in solanaceous and other crop species for adaptability to various stress conditions.

## Supplementary Materials

Supplementary materials can be found at https://www.mdpi.com/1422-0067/21/1/26/s1.
