# Supplementary material for: CaDHN4, a Salt and Cold Stress-Responsive Dehydrin Gene from Pepper Decreases Abscisic Acid Sensitivity in Arabidopsis
Source: Int J Mol Sci. 2019 Dec 19;21(1):26. doi: 10.3390/ijms21010026 (PMC6981442; doi:10.3390/ijms21010026)
Supplement: Supplementary file 1 [file ijms-21-00026-s001.pdf]

**supplementary materials:**

**Figure S1**

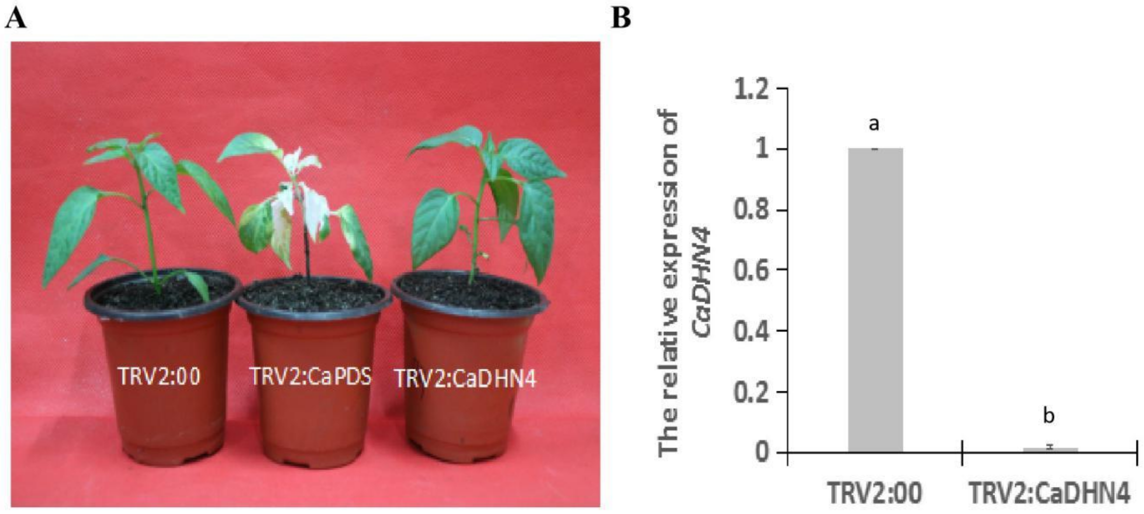


**Figure S1.** The phenotypes and analysis of *CaDHN4* expression of silencing pepper plants A: The phenotypes of silencing pepper plants; B: The relative expression of *CaDHN4* of silencing pepper plants.

**Figure S2**

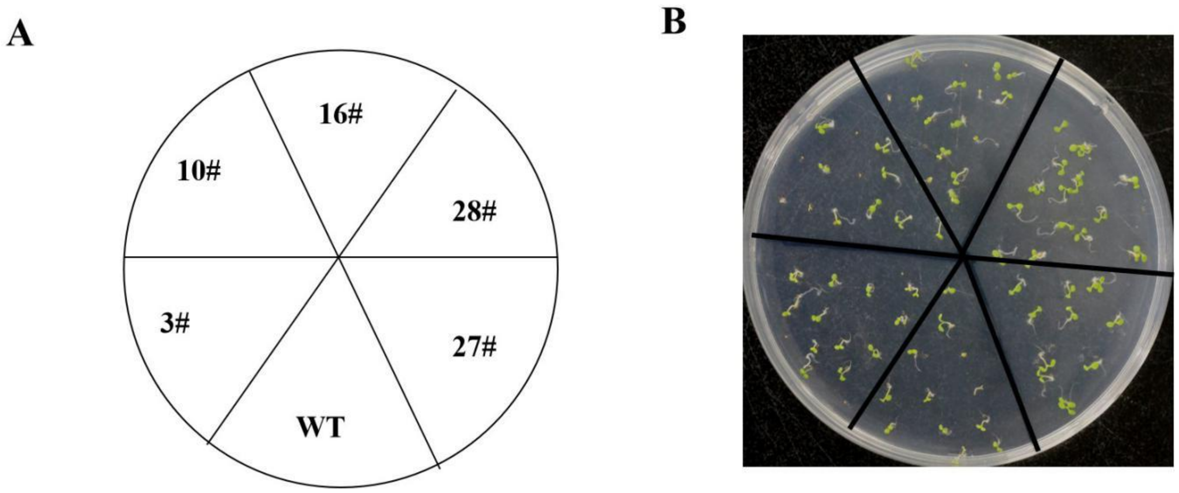


Figure S2 The phenotypes of *CaDHN4*-overexpressing plants under 0.5 μM ABA treatment

**Table S1.** sequence characteristics of *CaDHN4*

| Name | Gene ID | Chr | AA | MW (kDa) | PI | Introns | Localization predicted |
|---|---|---|---|---|---|---|---|
| *CaDHN4* | CA02g22060 | 2 | 172 | 18.44 | 7.3 | 0 | Nucleus |

Table S1. List of *CaDHN4* gene identified in pepper and sequence characteristics. Chr: chromosome; AA: amino acid; MW: molecular weight (kDa); pI: isoelectric point. Sequenced ID is from CM334 genome.

**Table S2.** The primer sequences of qRT-PCR.

| **Primer Name** | **Primer Sequence（5′—3′）** |
|---|---|
| AtDREB2A-F | ATGGCAGTTTATGATCAG |
| AtDREB2A-R | TCATACAACCCTTCTTCGA |
| AtCOR47-F | CAGTGTCGGAGAGTGTGGTG |
| AtCOR47-R | ACAGCTGGTGAATCCTCTGC |
| AtRD29B-F | CCCACGCATAAAGGTGGAGA |
| AtRD29B-R | AACTCATGGCTTCTCGTCGG |
| AtERD7-F | ATCTCTTCCCTGAACAACCA |
| AtERD7-R | GCGAGAACAGCGACAACAT |
| AtNCED3-F | ACATGGAAATCGGAGTTACAGATAG |
| AtNCED3-R | AGAAACAACAAACAAGAAACAGAGC |
| AtABF3-F | CTGATACAGACGCAGGAGAGG |
| AtABF3-R | AGGAACAGGGGACAAAGATG |
| ACTIN2-F | GGTAACATTGTGCTCAGTGGTGG |
| ACTIN2-R | AACGACCTTAATCTTCATGCTGC |
